# P-820. Analyzing Trends in Candidemia: Insights from Routine Susceptibility Testing

**DOI:** 10.1093/ofid/ofaf695.1028

**Published:** 2026-01-11

**Authors:** Nicholas M Moore, Mary L Scorza, Maria D Jimenez Macias, Patricia Mangan, Allison Wells, Jyothi Cheerala, Gregory Bragg, Hayley A Hodgson, Fischer Herald, Joyce H Houlihan, Shivanjali Shankaran, Sarah Y Won

**Affiliations:** Rush University Medical Center, Chicago, IL; Rush University Medical Center, Chicago, IL; Rush University Medical Center, Chicago, IL; Rush University Medical Center, Chicago, IL; Rush University Medical Center, Chicago, IL; Rush University Medical Center, Chicago, IL; Rush University Medical Center, Chicago, IL; Rush University Medical Center, Chicago, IL; Rush University Medical Center, Chicago, IL; Rush University Medical Center, Chicago, IL; Rush University Medical Center, Chicago, IL; Rush University Medical Center, Chicago, IL

## Abstract

**Background:**

Candidemia is associated with significant morbidity and mortality and rising healthcare costs. Echinocandins are recommended for empiric therapy. The aim of this study was to describe temporal trends in antifungal susceptibility results in *Candida* species to inform empiric treatment and antimicrobial stewardship efforts.

**Figure:**
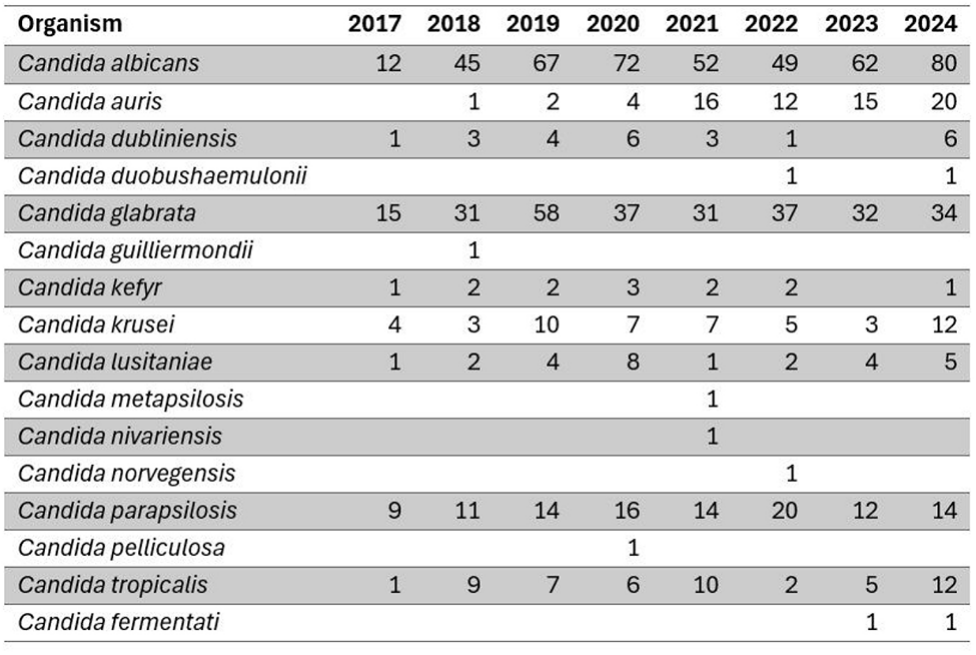
Candida isolates by year Counts of Candida species isolates identified each year.

**Figure:**
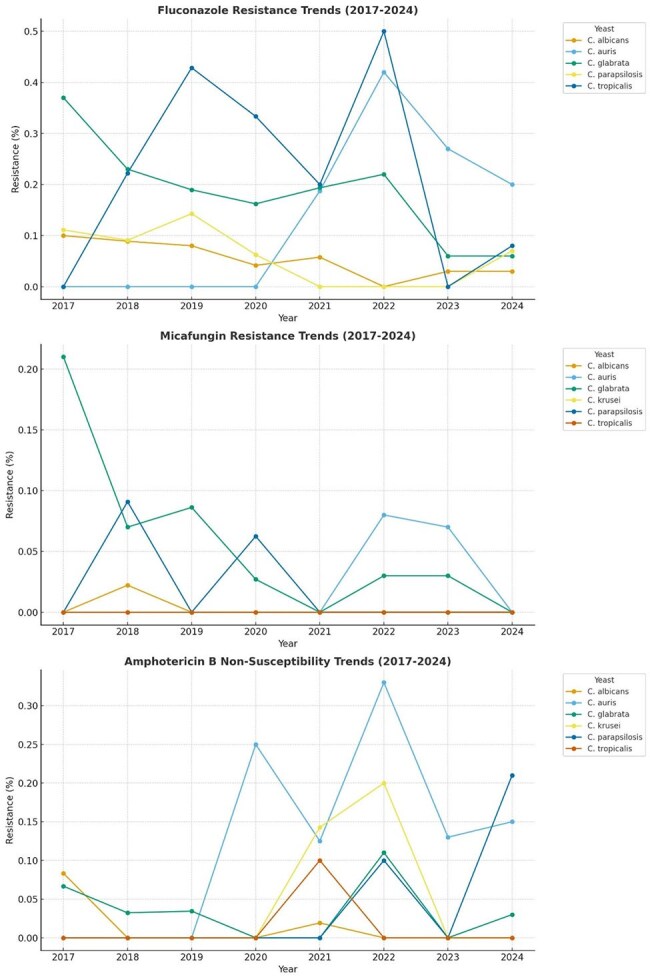
Candida species resistance trends over time Antifungal Resistance Trends in Fluconazole, Micafungin and amphotericin B among select Candida species.

**Methods:**

All non-duplicate *Candida* bloodstream isolates identified between May 11, 2017 through December 31, 2024 from a three-hospital healthcare system in Chicago were included. Isolates were identified by MALDI-TOF MS (Vitek MS, bioMérieux). Antifungal susceptibility testing was performed using an MIC method (YeastOne, Thermo Fisher). Minimal inhibitory concentrations (MICs) were interpreted using current CLSI breakpoints or epidemiologic cutoff values (ECVs) outlined in the M27M44SE and M57SE documents, respectively. Descriptive statistics were used to summarize microbiology data over time. The Cochran-Armitage trend test was used to analyze resistance and non-susceptibility to antifungal agents over time.

**Results:**

Over the period examined, we tested 1,070 *Candida* isolates. *Candida albicans* (439, 41%) was identified most frequently, followed by *C. glabrata* (275,25.7%)*, C. parapsilosis* (110, 10.3%), *C. auris* (70, 6.5%), *C. tropicalis* (52, 4.9%) and *C. krusei* (51, 4.8%). The remaining 73 isolates were other *Candida* species (Table). We evaluated trends in resistance to three antifungal agents routinely reported: fluconazole (FLC), micafungin (MCF) and amphotericin B (AMB) (Figure). Among *C. auris,* there was a statistically significant increase in FLC (p=0.048) and MCF (p< 0.001) resistance over time. *C. krusei* (p=0.028) and *C. parapsilosis* (p=0.002) had statistically significant increases in AMB non-susceptibility, indicating more isolates with non-wild-type MICs over time.

**Conclusion:**

In our laboratory, we observed a significant increase in the frequency of isolation of *C. auris* over time. Additionally, we observed an increase in FLC and MCF resistance in *C. auris* isolates over time and an increasing number of *C. krusei* and *C. parapsilosis* isolates with MICs to AMB above the ECV. These findings are concerning and warrant further investigation and collaboration with antimicrobial stewardship.

**Disclosures:**

All Authors: No reported disclosures

